# Competing visual flicker reveals attention-like rivalry in the fly brain

**DOI:** 10.3389/fnint.2012.00096

**Published:** 2012-10-19

**Authors:** Bruno van Swinderen

**Affiliations:** Queensland Brain Institute, The University of QueenslandBrisbane, QLD, Australia

**Keywords:** Drosophila, selective attention, electrophysiology, virtual environments, steady state visually evoked potentials (ssVEP), visual perception

## Abstract

There is increasing evidence that invertebrates such as flies display selective attention (van Swinderen, 2011a), although parallel processing of simultaneous cues remains difficult to demonstrate in such tiny brains. Local field potential (LFP) activity in the fly brain is associated with stimulus selection and suppression (van Swinderen and Greenspan, 2003; Tang and Juusola, 2010), like in other animals such as monkeys (Fries et al., 2001), suggesting that similar processes may be working to control attention in vastly different brains. To investigate selective attention to competing visual cues, I recorded brain activity from behaving flies while applying a method used in human attention studies: competing visual flicker, or frequency tags (Vialatte et al., 2010). Behavioral fixation in a closed-loop flight arena increased the response to visual flicker in the fly brain, and visual salience modulated responses to competing tags arranged in a center-surround pattern. Visual competition dynamics in the fly brain were dependent on the rate of pattern presentation, suggesting that attention-like switching in insects is tuned to the pace of visual changes in the environment rather than simply the passage of time.

## Introduction

Attention describes our ability to focus our perception on one stimulus (or group of related stimuli), while filtering out other simultaneous stimuli that are less relevant at any moment (Posner et al., 1980). A fundamental feature of attentional processes is that they involve parallel processing, whereby competing stimuli are simultaneously selected or suppressed. In this study, I use the fly, *Drosophila melanogaster*, to investigate visual competition dynamics in a small insect brain.

The evidence for selective attention in insects stems mostly from visual studies in behaving animals (Spaethe et al., 2006), from tethered paradigms in which visual stimuli can be more carefully controlled (Sareen et al., 2011), and finally from electrophysiological preparations in which neural correlates of visual attention can be measured [see (van Swinderen, 2011a) for a recent review]. Local field potential (LFP) recordings from the fly brain have revealed selection and suppression of 20–50 Hz responses to visual stimuli presented in opposition to either eye, either as competing objects 180° apart rotating around a tethered fly (van Swinderen and Greenspan, 2003; van Swinderen, 2007a; van Swinderen and Brembs, 2010), or as competing gratings presented to either eye (Tang and Juusola, 2010). Whether flies can attend selectively to simultaneously-presented visual stimuli in the same visual field is a more difficult problem, because it is not obvious how to measure differential responsiveness to competing objects that are close together or overlapping. The closed-loop *Drosophila* flight arena provides a potential method for investigating attention-like processes, by for example, measuring differential behavioral responses to layered objects in a figure-ground arrangement (Heisenberg and Wolf, 1984). An alternate approach that has been successful in human attention research is to tag competing visual (Andersen et al., 2008), auditory (Muller et al., 2009), or tactile (Bardouille et al., 2010) stimuli with distinct frequency components that can be simultaneously tracked in brain activity. There is compelling evidence from human and animal studies that attention increases the coherence and/or amplitude of attended frequency tags and the objects that they represent (Vialatte et al., 2010), although the neural mechanism for this form of gain (or phase) control remains unknown. In this study, I combine these two approaches (closed-loop fixation behavior and frequency tags) to explore the effect of competing visual flicker on fly brain activity, and find that behavioral fixation and novelty-induced salience both increase the power of frequency tags in the fly brain. Visual novelty evokes a stereotypical alternation dynamic between the competing tags that is dependent on how fast the tagged objects are moving.

## Materials and methods

### *D. melanogaster* strains and stocks

Flies were cultured at 22°, 50–60% humidity, 12 h:12 h light:dark cycle on standard media. Flies were raised at low density in bottles to promote flight behavior. Wild-type flies are from the Canton-S strain (CS); only 2–7 days-old flies were tested, and only cold anesthesia was used to prepare flies for tethering. Male flies were tested, unless specified otherwise.

### Preparation of flies for electrophysiology

**Figure A1:**
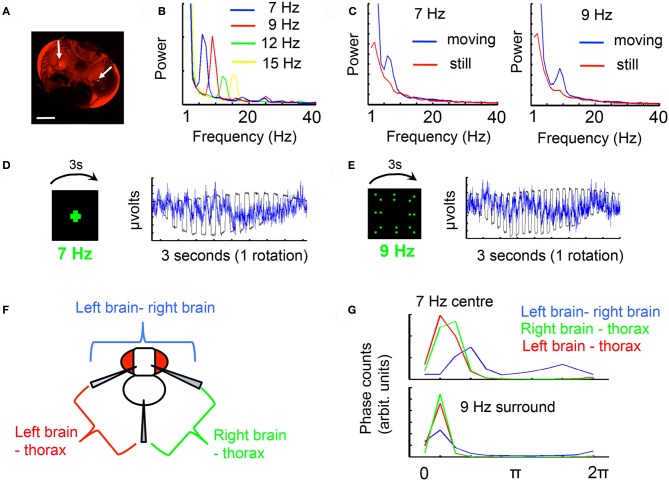
**The frequency tag. (A)** Recording sites in the *Drosophila* brain (arrows). Texas Red dye was released from glass recording electrodes by iontophoresis, and the two recording sites determined by the region with greatest staining intensity, in the inner optic lobe. A differential recording was made between these sites. Inset scale bar = 100 μm. **(B)** A moving object (a cross moving 120°/s) flickering at different frequencies evokes distinct frequency responses in the recorded brain LFP, shown here for four different frequency tags. **(C)** A moving flickering object (a cross moving 120°/s) evokes a greater frequency response in the brain LFP than a static flickering object (positioned 45° to the left of the front of the fly) for either 7 or 9 Hz. **(D)** The LFP tag (blue trace) is time-locked to a cross flickering at 7 Hz (black trace). The response for a full rotation of the stimulus is shown. **(E)** The LFP tag (black trace) is time-locked to a field of dots flickering at 9 Hz (black trace). The response for a full rotation of the stimulus is shown. **(F)** Differential recordings to the thorax reveal the contribution from either optic lobe. **(G)** Coherence analysis (see “Materials and Methods”) between each differential recording (from **F**) and the physical stimulus (as in **D** and **E**, recorded with a photodiode) reveals a distinct profile for the combined brain recording (blue), vs. each single optic lobe references to the thorax (red and green). Coherence to the signal is weaker for the within-brain differential, indicating a different quality response than for each individual optic lobe. Data in the paper are all voltage differentials within the brain (blue).

Flies were anaesthetized on a 2°C cold block controlled by a Peltier element. Flies were secured to a tungsten wire, as described previously (van Swinderen, 2011b). The tungsten wire ended in a small hook. A small drop of dental cement (SynergyFlow A3.5/B3, Coltene Whaledent) was applied to the tungsten hook, and contact was made (using a micromanipulator) with the front/top of the thorax and the top of the head. The dental cement was cured with blue light, using a dental gun (SDI radii plus, Henry Schein Dental). Flies were then removed from the cold block and allowed to recover before electrodes were implanted. Glass electrodes (1.0 mm borosilicate with filament, World Precision Instruments) were made, implanted, and secured as described previously (van Swinderen and Greenspan, 2003; van Swinderen, 2011b) with a few modifications: the positioning of the tungsten hook along the center (front to back) of the flies' head allowed for an electrode to be implanted on either side at the dorsal rim of the eye. Electrodes were lowered (using a Narishige MM1000 micromanipulator holding a pair of forceps (van Swinderen, 2011b) ~100 μm into the fly head at a 45° angle from vertical, to ensure that the electrode tip ended up in the inner optic lobes on either side. Electrodes were secured in place with dental cement such that they were free standing. Position of the recording site was verified by releasing Texas Red dye by iontophoresis, fixing the head in 2% paraformaldehyde following an experiment, and visualizing under fluorescence microscopy (See Figure A1). Electrode positioning was accurate enough to ensure a reliable LFP in response to visual flicker (see below).

### Electrophysiology

Brain recordings were performed exactly as described previously (Nitz et al., 2002; van Swinderen, 2007b). Teflon-coated 25 μm tungsten wires were inserted into the electrolyte solution in either glass electrode; these wires were connected to a differential amplifier (Warner Instruments DP301) via a field-effect transistor connection (NBLabs). The recording is a voltage differential between the two electrodes, probably representing differential field effects produced by populations of optic lobe neurons near each electrode tip, as also shown in another recent study of LFPs in *Drosophila* (Tang and Juusola, 2010). Spectral analyzes of brain activity data were performed in Matlab by Fourier analysis of data sampled at 300 Hz.

### Closed-loop flight control

**Figure 1:**
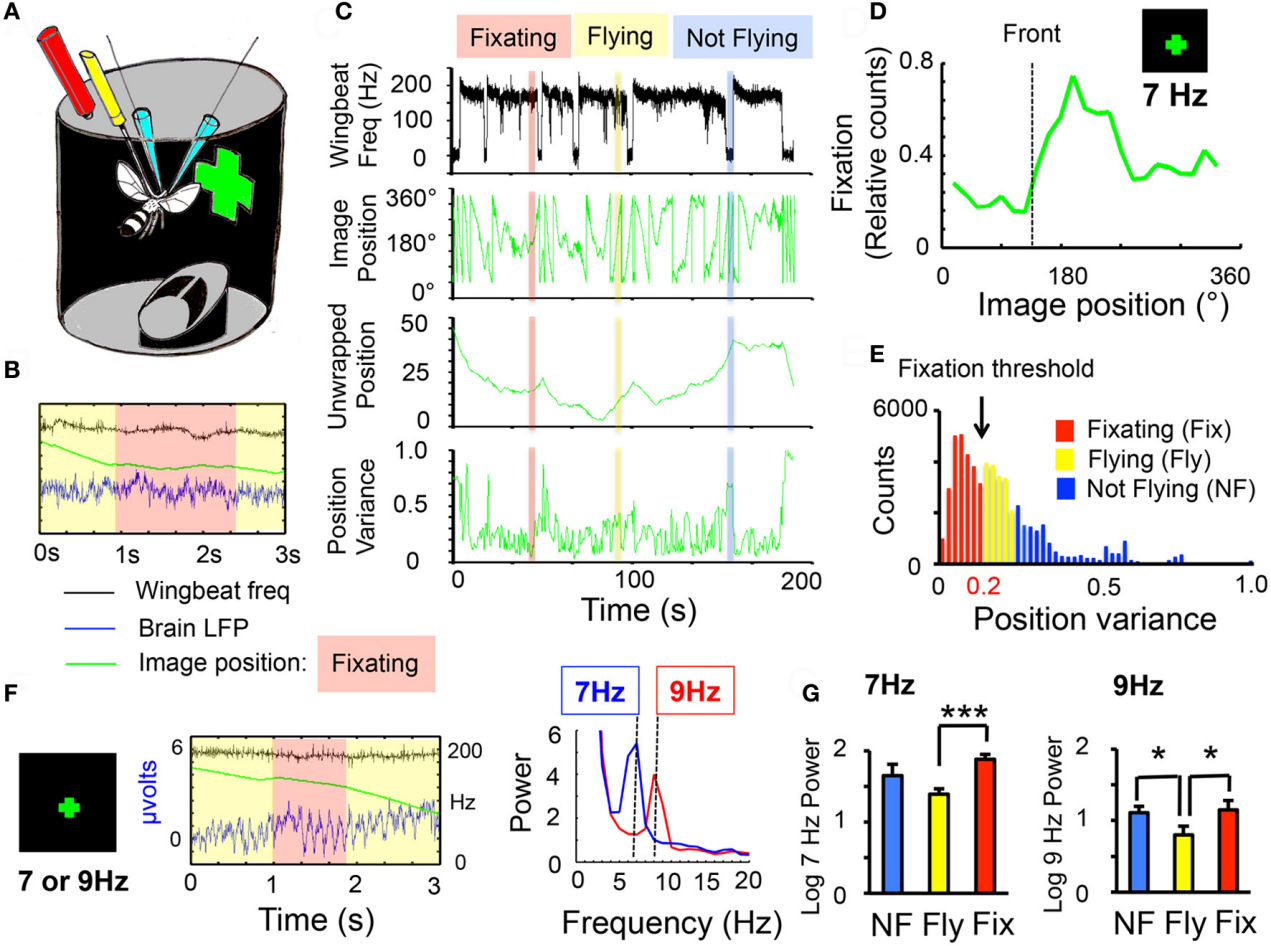
**Behavioral fixation of frequency-tagged visual stimuli. (A)** Experimental setup. Two glass electrodes (blue) are implanted into the brain of a fly tethered to a metal post (yellow). An infrared system (red) allows the fly to control the angular position of virtual objects (a green cross). **(B)** Three signals are recorded from each experiment: the wing-beat frequency (black, scale is 0–200 Hz), the local field potential (LFP, blue, scale is—2 to 6 μvolts), and the angular position of the image (green, scale is 0–360°). Behavioral fixation is observed when the fly stabilizes the angular position of the image (red shading). **(C)** Three behavioral states were identified (see “Materials and Methods”): Fixation (red), flight without fixation (yellow), and not flying (blue). Upper trace (black): wing-beat frequency, indicative of flight at 200 Hz. Lower trace (green): image position for the same recording. Unwrapped image position is shown below (to eliminate edges), and variance for the unwrapped data, below that. **(D)** Flies can fixate on a 30°-wide cross, composed of 20 LED pixels flickering together at 7 Hz. Image fixation is indicated by increased duration (relative time) in one part of the visual field, usually close to front (dotted line). Data are averaged from 6 wild-type male flies. **(E)** Fixation behavior was determined empirically per fly from the position variance statistics (see “Materials and Methods”). **(F)** LFP responses to a 7 or 9 Hz flickering cross. Middle panel: LFP (blue trace) recorded during fixation of a 9 Hz flickering cross. Green line: angular position of image (same axes as in C, second panel down); black trace: wing-beat frequency indicating flight. Fixation time is indicated by the red rectangle. Right panel: sample spectral analysis (see “Materials and Methods”) for a 7 Hz (blue) or a 9 Hz (red) cross during behavioral fixation. **(G)** Normalized 7 or 9 Hz power (log score ± SEM) for the three behavioral states (NF, blue, not flying; Fly, yellow, flight without fixation; Fix, red, flight with fixation, *n* = 6 male wild-type flies; ^*^P < 0.05, ^***^P < 0.001 by *t*-test). All responses were significantly greater than zero (= the 8 Hz denominator, indicating the presence of a frequency tag, *P* < 0.001).

The tethered fly, implanted with two electrodes, was able to control the angular position of virtual objects displayed on an LED arena by modulating its flight behavior, as described previously (Lehmann and Dickinson, 1997; van Swinderen and Greenspan, 2003). Briefly, an infrared light above the fly creates a shadow of either wing (in flight) on two photodetectors positioned below the fly (see Figure 1A). Wing beats are detected as a standing wave, and a differential of the wing beat amplitudes represents the fly's attempt at steering. Feedback from the wing beat differential to the LED arena controls the angular position of a visual panorama on the arena, such that flies are able to fixate on virtual objects, and this tendency to fixate is recorded as a position signal (from 1 to 72, sampled at 300 Hz) through time.

### Data analysis and statistics

Behavioral and electrophysiological data were analyzed offline with Matlab software. Custom-made code was used to identify behavioral states in the arena (fixation in flight, no fixation in flight, no flight). Flight was identified by a ~200 Hz signal from the wing beat analyzer. Behavioral fixation was determined from the image position information. Image position data (sampled a 300 Hz) was unwrapped to eliminate edges in the 1–72 positions (proportional to a 0–5.5 V signal) of the visual display. The degree of change through time in the unwrapped position data was then determined by calculating the variance of 1 s of position data, and moving this window across a time series, 1/300 steps at a time. This variance vector established a description of how fast the image was moving, and when it was momentarily stabilized. A frequency histogram of the position variance data for each fly revealed a variance distribution associated with behavioral fixation for each experiment. The variance threshold for fixation was thus determined from this distribution, which was empirically determined for each fly. In general, thresholds for fixation were between 0.15 and 0.3, with higher variances being indicative of flight without fixation (e.g., a permanent bias to one side), or no flight (open loop). LFP activity was also sampled at 300 Hz, and Fourier analysis of the data (Nitz et al., 2002) (using custom Matlab software) revealed the power associated with each frequency component. To normalize power for each fly prior to averaging across flies, power at each frequency tag (7 and 9 Hz in this study) was divided by 8 Hz power, which was not affected by the frequency tags, falls exactly between the two tags, and therefore represents an appropriate baseline LFP activity level for tag power in each fly. All these data were plotted on a log scale, such that responses above zero indicate an increased response, compared to 8 Hz. For some experiments, tags were compared as a log-ratio of 7 and 9 Hz, and these were zero-meaned (the average log-ratio was subtracted) so that responses above zero indicate increased power assigned to 7 Hz, and below zero indicates increased power to 9 Hz. Fourier analysis of defined temporal segments (defined by behavioral state, or by a novelty event, see below) was done in 1 s segments with a 0.75 s overlap, unless stated otherwise. Coherence between the LFP and the physical signal was calculated in Matlab by the Hilbert transformation of signals filtered for 7 or 9 Hz, using a Butterworth 4 filter. Statistics were performed in Matlab. Comparisons between conditions for were done by *t*-test (or U-test for non-parametric data, determined by the lilliefors test for normality). For experiments testing effects across time (which are often correlated in adjoining time bins), *P* values were corrected for multiple comparisons by applying the false discovery rate method (Benjamini and Hochberg, 1995).

### Visual stimuli and salience effects

**Figure 2:**
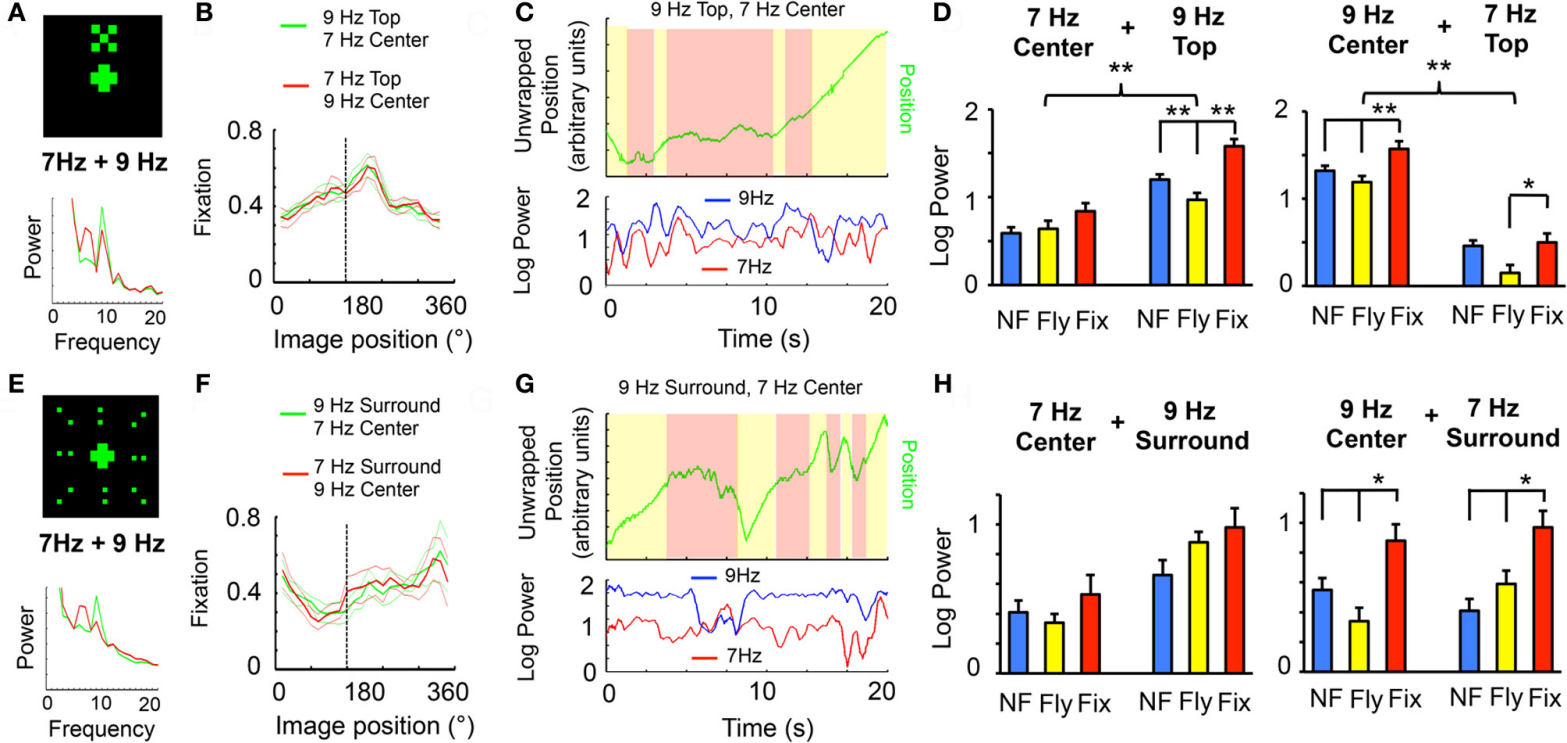
**Visual competition. (A)** A 30°-wide “X,” composed of 20 LED pixels flickering together at 7 or 9 Hz, was positioned above a centrally-positioned cross (as in Figure 1), also flickering at 7 or 9 Hz. Sample power spectrum is shown in the lower panel (green, 9 Hz top, 7 Hz center; red, reversed tags). **(B)** Flies were able to fixate on this compound object (dashed line shows frontal position), in either frequency combination (red vs. green, as indicated). **(C)** Ongoing analysis of 7 and 9 Hz power during fixation in a sample fly. **(D)** Left panels: Log-normalized 7 or 9 Hz power (± SEM) for the three behavioral states (NF, blue, not flying; Fly, yellow, flight without fixation; Fix, red, flight with fixation) when 7 Hz is center and 9 Hz is top. Right panels, the same with swapped frequency tags. *n* = 6 male wild-type flies; ^*^P < 0.05, ^**^P < 0.01, ^***^P < 0.001 by *t*-test. 9 Hz and 7 Hz groups were significantly different (*P* < 0.01, by ANOVA), as indicated (upper brackets). The same flies contributed to both sets of data (left and right panels). **(E)** The cross surrounded by a field of 16 LED dots, subtending 90°, flickering at 7 or 9 Hz. Sample power spectrum is shown in the lower panel (green, 9 Hz surround, 7 Hz center; red, reversed tags). **(F)** Flies were able to fixate on this compound stimulus (dashed line shows frontal position), in either frequency combination (red vs. green, as indicated). **(G)** Ongoing analysis of 7 and 9 Hz power during fixation in a sample fly. **(H)** Left panels: Log-normalized 7 or 9 Hz power (± SEM) for the three behavioral states (as in D) when 7 Hz is center and 9 Hz is the surround. Right panels, the same with swapped frequency tags. *n* = 6 male wild-type flies; ^*^P < 0.05, by *t*-test. The same flies contributed to both sets of data (left and right panels).

Visual stimuli were controlled using custom-written Labview software (National Instruments), as described previously (van Swinderen and Greenspan, 2003; van Swinderen, 2007b). To create visual flicker, images alternated between a blank template of the 72 × 24 LEDs and a frame of the lit image for the object involved, and altering the delay for either frames controlled flicker rate. The visual stimulus was captured by a photodiode in the arena (See Figure A1), this signal was recorded at 300 Hz, and Fourier analysis of the signal confirmed the flicker frequency. Stationary flicker produced much less of a response (Figure A1), so was not used for this study. Different flicker frequencies could be superimposed on the same 72 × 24 LED panorama, thereby creating the dual-flicker compound objects used in this study. Since frequency combinations above 10 Hz proved unreliable (they did not produce those exact, separable frequencies in the LED arena, because of limitations in the Labview updates), a combination of 7 and 9 Hz was used for most experiments, unless specified otherwise (see Figure A1 for other frequency examples). More detailed spectral analysis of these separate and combined signals revealed the flicker rate to actually be 6.5 and 8.9 Hz. No differences in the results were found by simplifying the analyses to 7 and 9 Hz throughout the study. The stimulus used was either a “+” or an “×,” each subtending 30° (square) of the arena, and exactly 20 LED pixels. The cross or “×” was centrally positioned in the arena, such that it occurred in the fly's lower visual field (flies were angled ~20° up from horizontal). Competing stimuli consisted an “×” positioned over a “+” flickering at a distinct frequency (as in Figure 2A), or of 16 single pixels arranged around the central object (as in Figure 2E), also flickering at a distinct frequency. In the second scenario, the surround therefore created a 70°—wide window around the 30° central object. To study the effect of novelty and time, the central object alternated between a “+” and the “×,” while the surround remained unchanged. Alternation times were drawn from a random number generator, between 5 s and 50 s, and each experiment lasted approximately 700 s, during which on average ~25 changes occurred. Analyses of LFPs after a visual change were done only after a 100 ms delay, to prevent any flicker artifact associated with the switch between objects. These LFP data were contrasted (as a ratio) to data in the same frequency domain before a change, to determine whether there were any novelty salience effects.

### Brain imaging of recording sites

Localization of the recording sites was determined by releasing Texas Red dye via iontophoresis. An aliquot of 1 μl of dye was introduced into the recording electrode following a recording session, and allowed to dissipate throughout the recording solution. A pulsating current was applied to release the dye, via a 25 μm tungsten wire inserted into the relevant electrode and a reference in the thorax. Decapitated heads were then fixed in 2% paraformaledhye for 1 h, then moved to PBS solution, partially dissected to view the brain, and whole-mounted for fluorescence imaging. Recordings were from either optic lobe, in the vicinity of the lobula (see Figure A1).

## Results

### Behavioral fixation increases frequency tag power

Tethered flies in a closed-loop flight arena (Lehmann and Dickinson, 1997) are able to control the angular position of virtual objects (e.g., a green lit cross, see “Materials and Methods”) by modulating their wing-beat behavior (Figure 1A). Recordings of LFPs taken from the fly brain were combined with this behavioral paradigm in order to investigate neural correlates of visual fixation. Each experiment thus provided three simultaneous signals: (1) Wing-beat frequency, indicating flight, (2) LFP data, indicating the brain's response to visual stimuli, (3) Image position data, indicating the angular position of the virtual object, and thereby fixation behavior (Figure 1B). In the course of an experiment, flies could be in three distinct behavioral states: fixating while flying, flying but not fixating, or not flying—which can all be surmised from the wing-beat and image position signals (Figure 1C). To provide a temporal tag to the virtual object, the visual cue was made to flicker at a distinct frequency (e.g., a lit cross flickering at 7 Hz). Flies were able to fixate on the 7 Hz object, with fixation typically resulting in the flickering object being positioned (by the fly) transiently in the right visual field (Figure 1D). Fixation thresholds were determined empirically for each fly (Figure 1E, and see “Materials and Methods”), in order to investigate how changes in behavioral state affected LFP activity in response to the visual object. Frequency tags (7 or 9 Hz) were visible in the raw LFP, and were clearly distinct in spectral analyses of the data (Figure 1F). Previous work has shown that fixation on a non-flickering object increased LFP activity (van Swinderen and Greenspan, 2003). Consistent with that study, I found that fixation on a 7 or 9 Hz flickering object increased the power at that frequency compared to flight without fixation (Figure 1G, red vs. yellow histograms). Surprisingly, non-flying animals exposed to the moving flicker also displayed increased power at the tagged frequency compared to non-fixating flying animals (Figure 1G, blue vs. yellow histograms), although this was only significant for 9 Hz. This difference between flight without fixation and non-flight cannot be due to image exposure, because in both cases the object is rotating steadily around the fly. One explanation for this result might be that during flight without fixation, visual stimuli are somehow suppressed, compared to flight with fixation, or even the flightless condition.

### Competing frequency tags

Possessing two frequencies, 7 and 9 Hz, that evoked separable responses in the fly brain (Figure 1F), I combined these tags as distinct visual components of a compound pattern that the fly could potentially fixate, presenting the competing stimuli in a vertically aligned arrangement: either a 30°, 7 Hz central cross topped by a 9 Hz “X” of similar dimensions (see “Materials and Methods”), or the same with the frequencies swapped (Figure 2A). Flies were able to fixate on the compound 7 and 9 Hz object (Figure 2B). This arrangement allowed the following question to be asked: how is brain responsiveness partitioned between the competing tags during behavioral fixation? A spectrogram of 7 and 9 Hz power revealed that the competing stimuli represented in the brain LFP could be uncorrelated at times (Figure 2C). Spectral analysis for the three different behavioral states revealed increased responsiveness to the 9 Hz stimulus compared to 7 Hz, regardless of whether it was on top or below (Figure 2D). However, comparing behavioral states showed a similar trend as found earlier (Figure 1G) for objects presented individually: fixation tended to increase power of both frequency tags, together, compared to flight without fixation (Figure 2D).

A recent behavioral study using a similar closed-loop flight paradigm suggested that fly responsiveness to visual cues is unequally distributed between the upper and lower visual field (Sareen et al., 2011), which may account for differences in tag power depending on whether the lower (central) or upper object was represented by that frequency (Figure 2A). An alternative way of presenting visual competition that partially sidesteps the issue of upper/lower visual fields, is to surround a central flickering object with a uniform field of dots flickering at an alternate frequency: either a 30°, 7 Hz central cross surrounded by a 90° field of 9 Hz dots, or the same with the frequencies swapped (Figure 2E, and see Figure A1 for details on the stimulus). Flies were also able to fixate this larger, compound 7 and 9 Hz pattern (Figure 2F), although their fixation range was broader, probably because the entire visual covered 90°, any part of which could potentially be fixated. Analysis of fixation epochs revealed again that responses to the tags could be uncorrelated: power of the surrounding tag could be transiently decreased, and the central tag could be augmented (Figure 2G). Also, there was again a tendency for increased power for either tag during behavioral fixation, compared to flight without fixation (Figure 2H), although this was only significant when 9 Hz was central. The previously observed trend of lower responsiveness for flight alone (lower yellow histograms, compared to blue and red in Figures 1G, 2D) seems to be abolished when the tag is in the surround (Figure 2H, 7 Hz Surround and 9 Hz Surround), but maintained when the tag is central (Figure 2H, 7 Hz Center and 9 Hz Center). Together, these experiments show that multiple tags distributed in more complex patterns evoke similar responses as single tags for one object alone. This raises the question of whether flies can display differential responses to competing frequency tags.

### Novelty salience increases frequency tag power

**Figure 3:**
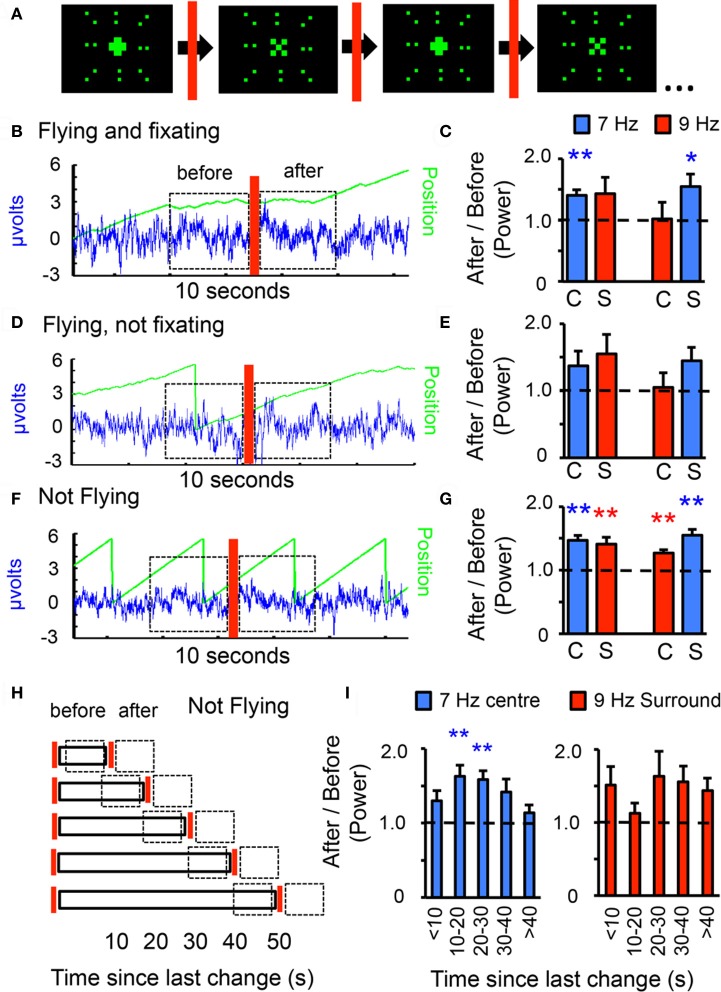
**Visual novelty modulates frequency tag power. (A)** Center-surround pattern, with visual changes in the center. The central object flickering at 7 or 9 Hz alternates between a 30° “+” and an “×,” with changes happening randomly every 5–50 s. The changing central object is surrounded by an unchanging 90° surround composed of dots flickering in synchrony at 7 or 9 Hz. **(B)** Epochs of behavioral fixation that included a visual change were analyzed for 7 and 9 Hz power. Power for either frequency 3 s after the change were contrasted, as a ratio, with 3 s before the event (dashed boxes). Blue trace: LFP; green line: image position, as in Figure 1C. **(C)** 7 and 9 Hz power after/before ratios for novelty events during fixation epochs, for either tag configuration. C, center; S, surround; *n* = 6 flies, ^*^P < 0.05, ^**^P < 0.01 by *t*-test compared to 1.0 (dashed line). **(D)** Novelty events occurring during flight epochs without fixation were analyzed for 7 and 9 Hz power before and after the visual change in the center. **(E)** 7 and 9 Hz power after/before ratios for novelty events during flight without fixation, for either tag configuration (*n* = same 6 flies as in C). **(F)** The compound 7 and 9 Hz pattern was moved around the fly with a period of 3 s (120°/s), with the center changing randomly (indicated by the red bar). LFP power for either frequency 3 s after the change were contrasted, as a ratio, with 3 s before the event (dashed boxes). Blue trace: LFP; green line: image position. **(G)** 7 and 9 Hz ratios for novelty events during non-flight epochs, for either tag configuration (C, center; S, surround). *n* = 12 flies, ^**^P < 0.01, by *t*-test compared to 1.0 (dashed line = no effect). **(H)** The same data as in G was reanalyzed after binning into five separate categories depending on how much time had passed since the last change. A ratio was calculated for either tag, contrasting the 3 s after vs. 3 s before (dashed boxes). **(I)** 7 Hz (blue) and 9 Hz (red) ratios for visual changes during non-flight experiments, binned into 5 temporal groups. Shown are results for 7 Hz center vs. 9 Hz surround. *n* = 12 flies, ^**^P < 0.01, by *t*-test compared to 1.0 (dashed line).

In previous work we have shown that visual salience, such as novelty, increases endogenous 20–30 Hz LFP activity in the *Drosophila* brain (van Swinderen and Greenspan, 2003; van Swinderen, 2007b; van Swinderen et al., 2009; van Swinderen and Brembs, 2010). To investigate whether such salience effects might change the responsiveness of flies to specific flicker frequencies, the preceding center-surround scenario was modified to include a changing central object, while the surround was left unchanged (Figure 3A). The central object alternated between a “+” and an “×” with random timing set between 5 s and 50 s. Power for either tag (7 or 9 Hz) after a change was compared to power before the change, expressed as a ratio, for either frequency (Figures 3B–G, see “Materials and Methods”). Changing the central object shape (but not the frequency tag) while the fly was actively fixating on the compound stimulus (Figure 3B, red bar) resulted in significantly increased 7 Hz power; effects on 9 Hz were less clear, although the tendency was for increased power following the change, regardless of the frequency tag (Figure 3C). Any significant effects were lost when novelty occurred (for the same animals) during flight without fixation (Figures 3D,E), although the trend to increased responsiveness was similar as for fixation, and these results were not significantly different from the novelty results under active fixation. In a separate set of open-loop experiments in non-flying animals (Figure 3F), changes to the central object shape also increased the power of the competing surrounding tag as well as the central tag, for either frequency combination (Figure 3G). This last, most significant set of results demonstrates that responsiveness to visual flicker can be modulated in the absence of flight behavior, and further, that a visual salience event (a novel object at the center) evokes increased responsiveness to a wider area than just the 30° central object that is changing. Together, the results so far suggest that novelty effects on the frequency tag may be independent of behavioral state of the animal. In addition, responsiveness to the competing tags 3 s after a change appears to be broad rather than selective. Since the novelty effect on tag power was also present in non-flying animals, subsequent analyses will focus on this more extensive dataset, specifically, looking at 7 Hz center competing with a 9 Hz surround (this combination produced a similar-sized novelty effect for both frequency tags, Figure 3G, left histograms).

### Novelty salience effects depend on stimulus parameters and elapsed time

Previous studies in non-flying *Drosophila* have shown that the magnitude of visual novelty effects depend on how much time has elapsed before an image changes (van Swinderen, 2007b; van Swinderen and Brembs, 2010). To investigate whether novelty salience effects on the frequency tags are also dependent on time elapsed before a change (changes occurred randomly between 5 s and 50 s), the above open-loop, non-flying data (Figure 3G) were subdivided into five temporal groups from <10 to >40 s, binned by how much time had passed since the last novelty incident (Figure 3H). Accounting for time elapsed before novelty presented a strikingly different result for the competing frequency tags: responsiveness to a changing 7 Hz central object was maximal when ~20 s had elapsed; more elapsed time (>40 s) failed to cause an increase in the central tag (Figure 3I, left panel). In contrast, the surrounding 9 Hz tag never reached significance, although 9 Hz power appeared uncorrelated to 7 Hz when partitioned this way (Figure 3I, right panel). This suggests that the competing frequency components do not contribute equally to the brain response (as might have been surmised from the combined data in Figure 3G), and that elapsed time determines what tag the fly brain is likely to respond to in the center/surround pattern.

**Figure 4:**
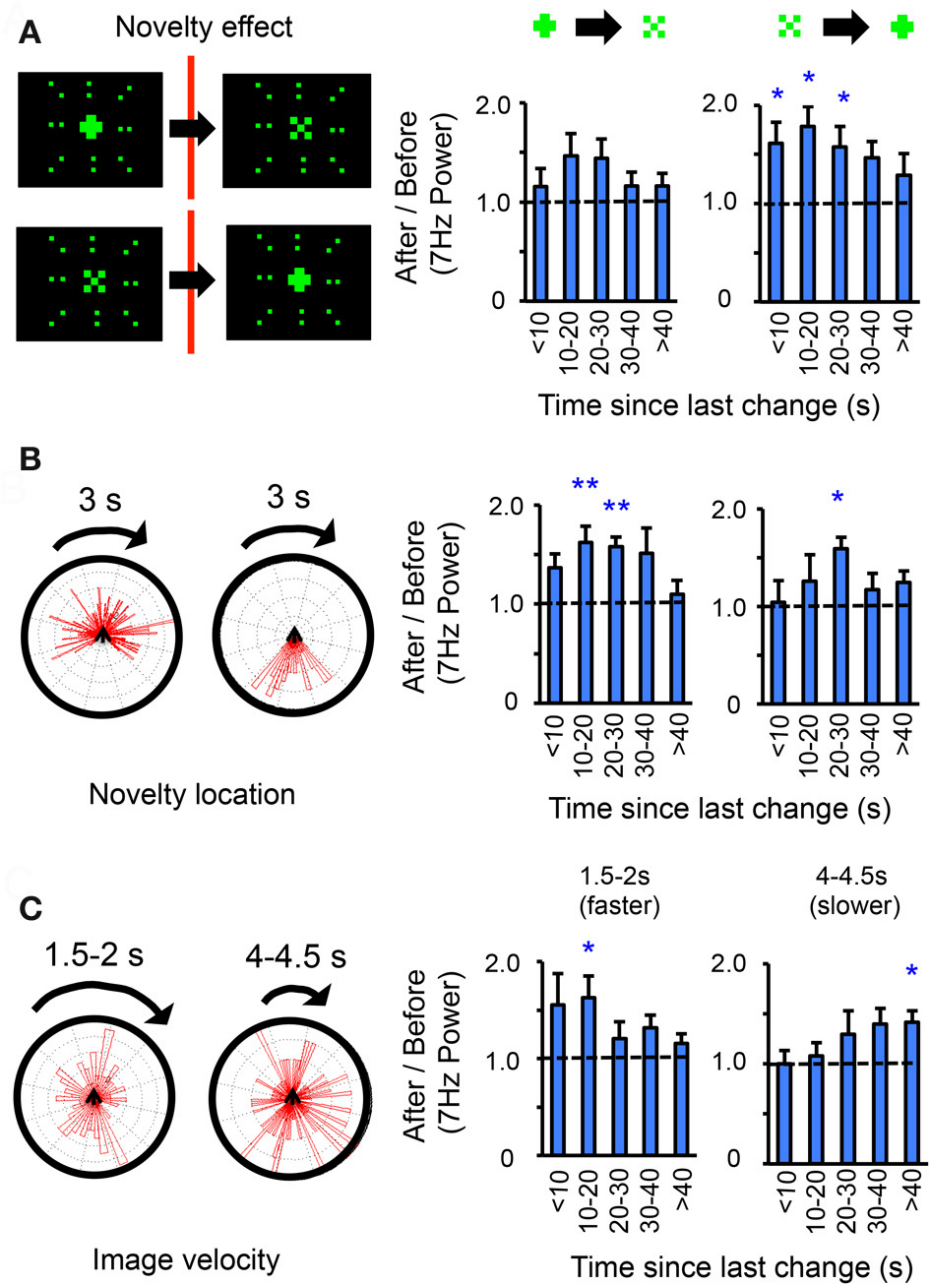
**Object shape, novelty location, and image velocity modulate visual salience effects. (A)** Left panels: Novelty could either be from a “+” to an “×” or vice versa. The size of either 7 Hz object is identical (30° square, 20 pixels), the surrounding 9 Hz display never changes. Right panels: the same data as in Figure 3G, partitioned into either transition sequence (indicated above the histograms). *n* = 12 flies, ^*^P < 0.05, by *t*-test compared to 1.0 (dashed line). Only 7 Hz effects are shown. **(B)** Effect of novelty location/timing. Left panels: the same data as in A was divided according to where in the rotation sequence changes occurred (in front of the fly, left rose plot; behind, right rose plot), and 7 Hz power ratios (3 s after/3 s before) recalculated for each situation (right panels), *n* = 12 flies, *P* < 0.05, ^**^P < 0.01, by *t*-test compared to 1.0 (dashed line). **(C)** 7 Hz ratios (3 s after/3 s before) for a faster moving pattern with a period of 1.5–2 s, or a slower moving pattern with a period of 4–4.5 s. *n* = 9 flies, ^*^P < 0.05, by *t*-test compared to 1.0 (dashed line). Rose plots on left indicate positions where changes occurred for either set of experiments.

Does object shape matter? Subdividing the same dataset further revealed a similar loss of responsiveness to the 7 Hz center after 40 s, regardless of whether change had been from a “+” to an “×” or *vice versa*, although only increased LFP responsiveness for a “+” was significant (Figure 4A). Previous studies have shown that vertical bars are attractive in a similar paradigm (Maimon et al., 2008), which may explain the increased response to the “+,” which includes a vertical component. A different question was whether the novelty salience effects depended on where changes happened in the fly's visual field. For example, a change occurring behind the fly (where it cannot be seen) might not be as salient as a change happening in front of the fly, which might be startling. This appeared to be the case, although the peak novelty effect (at ~20 s elapsed time) was still significant (*P* < 0.05) for changes occurring behind the fly (Figure 4B), suggesting that this is not entirely a startle phenomenon and that the fly may be primed to respond to changes at the center of the visual display after 20 s. Together, these more detailed analyses of one frequency tag (7 Hz center) indicate that responsiveness in the brain LFP depends on elapsed time between novelty events.

### Absolute time vs. stimulus time

Why are wild-type *Drosophila* brains responsive to visual changes after ~20 s but not after 40 s in this scenario? One possibility is that it is not absolute time that is critical here, but rather the stimulus presentation rate in this particular paradigm. In the open-loop (non-flying) scenario, the 7 and 9 Hz visual pattern rotates around the fly once every 3 s, or 120°/s (Figure 3F, and see “Materials and Methods”). To test whether the pattern speed determined when flies were most responsive to change, the same experiments were performed with the patterns sped up or slowed down (Figure 4C). In the faster scenario (1.5–2 s, or 240–180°/s), significant responses occurred after less elapsed time (10–20 s), whereas in the slower scenario (4–4.5 s, or 80–90°/s), significant responses occurred after more elapsed time (40 s, Figure 4C). This supports the possibility that pattern speed, rather than absolute time, determines the differential responses to the central object.

**Figure 5:**
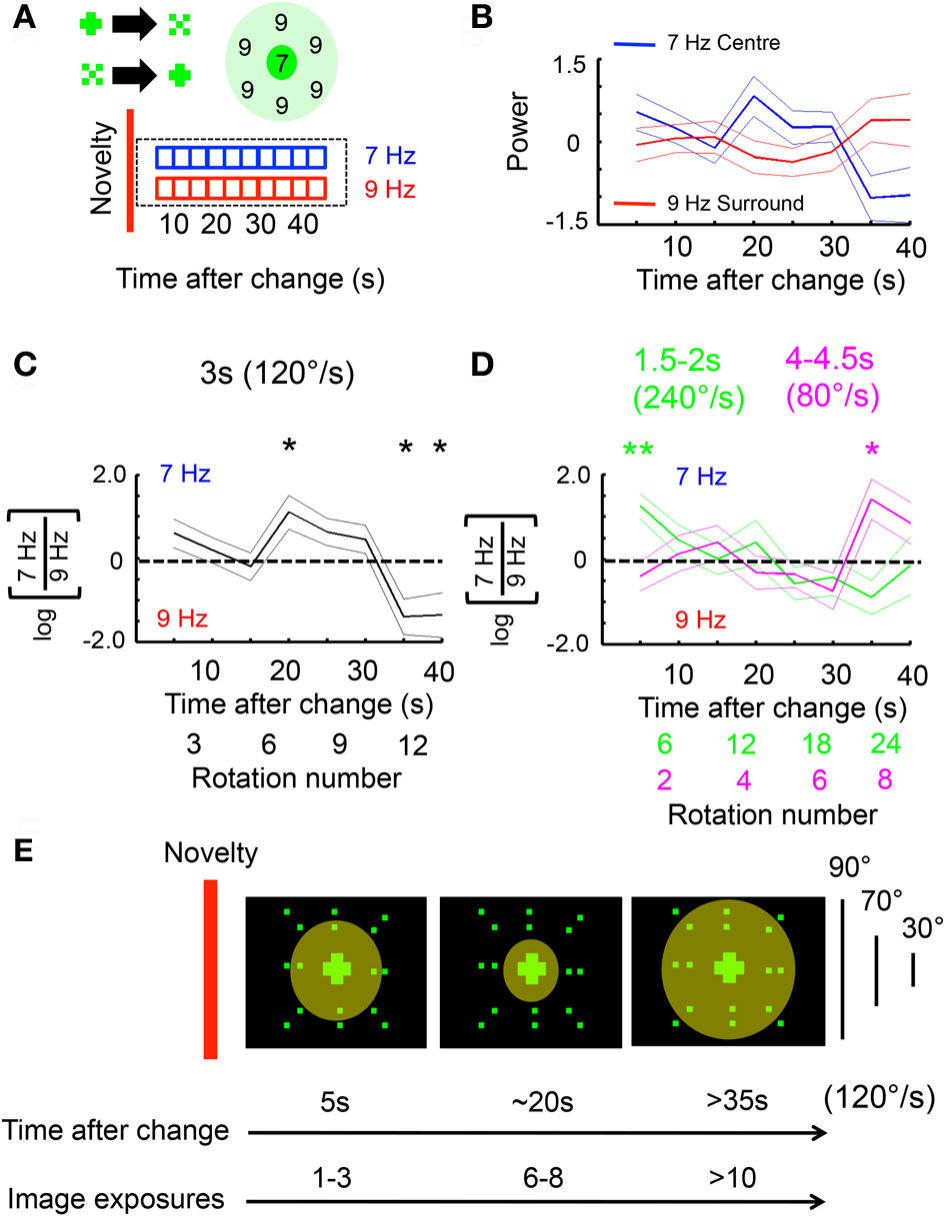
**Ongoing frequency tag dynamics are tied to pattern velocity. (A)** 7 Hz (center) and 9 Hz (surround) power was calculated for a 3 s, moving window following a visual change, for an uninterrupted 40 s (see “Materials and Methods”). **(B)** Average log-normalized and zero-meaned 7 and 9 Hz power (± SEM) plotted over time, following a novelty event (*n* = 12 male flies). **(C)** Average log-ratio of 7 Hz/9 Hz (± SEM) for the same data as in B. ^*^P < 0.05, by *t*-test compared to zero (dashed line; greater 7 Hz power is above the line, greater 9 Hz below). The number of rotations is indicated below the time axis. **(D)** The same 7/9 Hz log-ratio analyses were performed on faster (green) and slower (magenta) moving objects. ^*^P < 0.05, ^**^P < 0.01, by *t*-test compared to zero. The approximate number of rotations for either image velocity condition is indicated by matching color below the time axis. **(E)** A model of the spatio-temporal dynamics of LFP frequency tag effects following a visual change in the center (red bar). The diameter of the circle indicates the size of a hypothesized attention “spotlight.” The corresponding number of image exposures at different times is indicated for an object moving around the fly at 120°/s. The angle subtended by the different object components (surround, area inside surround, and center) is indicated.

**Figure A2:**
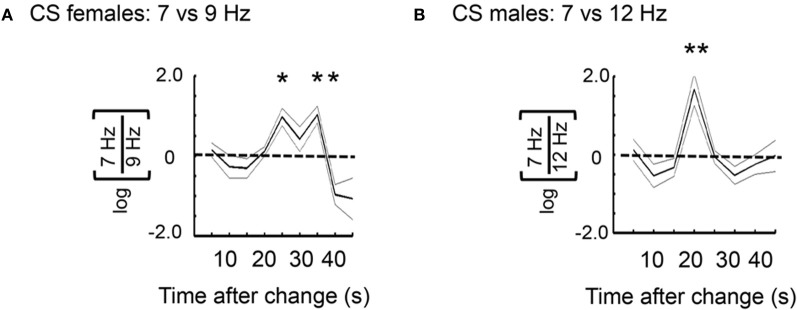
**Frequency tag dynamics in females, and in relation to another tag frequency in the surround. (A)** Log-ratio for 7 Hz center vs. 9 Hz surround, for 40 s after novelty (*n* = 4 female flies, ^*^P < 0.05, by *t*-test compared to 0, dashed line). **(B)** Log-ratio for 7 Hz vs. 12 Hz surround, for 40 s after novelty (*n* = 5 male flies, ^*^P < 0.01, by *t*-test compared to 0, dashed line).

In the preceding analyses I contrasted LFP responses to competing flicker before and after a visual change. I next investigated ongoing responses to competing flicker following a visual change. Ongoing 7 and 9 Hz power was calculated and averaged among flies (see “Materials and Methods”) across 40 s of time in a subset of recordings from the 3 s (120°/s) open-loop dataset (Figure 5A). Only recordings lacking a visual change until >40 s were investigated (about 5 of these occurred, by chance, per experiment). These analyses thus queried what happened to 7 and 9 Hz power during the entire 40 s after the last change. Surprisingly, brain responses to the competing tags over 40 s time were highly stereotypical, alternating between favoring the 7 Hz center around 20 s and the 9 Hz surround at 40 s (Figure 5B). A log-ratio analysis of the same data (rather than normalizing each frequency separately, see “Materials and Methods”) shows qualitatively the same result, with significant effects at 20 s in favor of 7 Hz and at 35–40 s in favor of 9 Hz (Figure 5C). Similar tag alternation effects were also seen in experiments on female flies, as well as in males exposed to a different competing frequency, 12 Hz (Figure A2). One interpretation of these results would suggest that when 7 Hz is dominant, flies are attending to the moving center, while suppressing the adjacent surround that is moving with it. Central to this interpretation is the notion that the fly is then actively attending to the flickering object as it moves, and therefore primed to respond at that time to any changes in the center.

To further investigate whether these attention-like effects are tied to the pattern presentation rate rather than absolute time, the same analyzes were done on experiments where the image rotation speed was slowed down (to a 4–4.5 s period, or 80–90°/s) or sped up (to a 1.5–2 s period, or 240–180°/s). As above, only long (>40 s) epochs without change were examined to capture the ongoing dynamics between the frequency tags, plotted as a log-ratio (Figure 5C). Increasing or decreasing image speed abolished the stereotypical 7 Hz increase seen at 20 s for the intermediate speed (Figure 5D). Instead, responsiveness for the 7 Hz tag peaked at 5 s for the fast-moving patterns, and at 35 s for the slow-moving patterns (Figure 5D). This suggests that, as for immediate novelty effects (Figure 4C), pattern presentation speed rather than absolute time was responsible for setting responsiveness levels to ongoing, competing visual flicker in the fly brain. A model of the 120°/s (intermediate speed) data would therefore suggest that selection of the central stimulus (and suppression of the surround) is greatest after ~6 exposures (rotations) of the compound stimulus (Figure 5E). This exposure-based interpretation is consistent with results from experiments performed at different pattern speeds (Figures 4C, 5D).

## Discussion

This study shows that LFP responses to visual flicker in the fly brain depend on behavioral state as well stimulus history and salience, suggesting that endogenous mechanisms in the fly brain are modulating synchronized neuronal responses to visual flicker. By presenting competing flickering stimuli to *Drosophila*, it should in principle be possible to study mechanisms of visual attention in the insect brain using paradigms traditionally used in human attention research. Indeed, the frequency-tagging approach used in this study to track attention-like behavior in flies was inspired from similar approaches applied to study human attention (Vialatte et al., 2010). Frequency tags (also termed steady-state visually evoked potentials, SSVEPs) provide common neurophysiological indices of attention that are equally accessible in both human subjects and animal models. However, the use of animal models allows more invasive techniques, such as genetic manipulation, to be applied in order to probe the underlying neurobiology of the response (Paulk and van Swinderen, in preparation). Surprisingly, SSVEPs have rarely been used to study visual attention in animals other than primates, possibly because few paradigms allow for brain recordings in restrained animals still capable of demonstrating visual-behavioral choices (Miller et al., 2011). One of the more striking results from the current study is that changes in frequency tag power in the fly brain associated with visual salience do not have to be associated with active behavior. Although behavior is crucial for supporting conclusions drawn about the relevance of frequency tags to attention, behavior is not a requirement for attention-like processes in human brains (Vialatte et al., 2010), and this seems to be true for insect brains as well, such as in *Drosophila*. We have come to a similar conclusion in previous work focused on endogenous oscillations associated with attention-like process in the fly (van Swinderen and Greenspan, 2003; van Swinderen, 2007a,b; van Swinderen et al., 2009; van Swinderen and Brembs, 2010); the current study extends this observation to exogenously-induced oscillatory activity.

The fact that attention (without any associated behavior) modulates visual responsiveness in human brain activity has been known and studied for a long time (Hillyard and Anllo-Vento, 1998; Vialatte et al., 2010), although it is not clear how such processes may become divorced from active behavior. That attention in animals may have evolved together with animal motility seems evident, if only as an anticipatory mechanism to avoid collisions, and this has also been written about extensively [see Rodolfo LLinas “I of the Vortex” (Llinas, 2002)]. Indeed, even simple animals such as flies show increased responsiveness of visual neurons when they are actively walking (Seelig et al., 2010) or flying (Maimon et al., 2010), although there has been little evidence that such changes in fly brain activity might be unequally distributed among competing visual stimuli (but see Tang and Juusola, 2010). In this study, I show that behavioral fixation in flies increases LFP responses to frequency-tagged visual stimuli, but that such effects can also be evoked in non-flying animals by visual changes, and that LFP responsiveness to competing stimuli appears to alternate in a rivalry-like manner. Visual salience and behavioral fixation effects support the idea that such frequency-tag alternations in the fly brain might be relevant to selective attention. These data suggest that wild-type flies are attending to restricted parts of their visual field at specified times, and hence more likely to detect any change occurring within the attended area at those times. One interpretation of these results is that fly attention resembles a “spotlight,” where cues outside of it are less likely to be perceived (Figure 5E).

In human studies, a spotlight metaphor is often used to describe attention processes (LaBerge, 1983), partially because that is how attention feels to our conscious minds. Recent *Drosophila* behavioral studies, in line with the current frequency-tag work, suggest that the fly attention “spotlight” might be quite wide [at least 40° (Sareen et al., 2011)], is mostly directed to the lower visual field (Sareen et al., 2011), and can be directed to one or the other eye in alternation (Tang and Juusola, 2010). Although tethered flight paradigms may not accurately reflect fly behavior in the wild, the frequency-tagging approach in the current study confirms that simultaneous visual cues are selected or suppressed in parallel in the fly brain, and shows that this “spotlight” effect follows a stereotypical spatiotemporal pattern in visual salience experiments. In wild-type flies, following a visual change, the selection window appears to encompass both tags (as seen in Figure 3G), suggesting that a wide area (at least 70°) is “attended”; however, after this salience effect, the spotlight appears to narrow and then widen again, for durations tied to stimulus exposure rates (Figure 5B). This alternation may represent a form of perceptual rivalry in the fly brain, where the focus of attention expands and contracts in accordance with the rate of change occurring around the animal.

Although stimulus rivalry might be under some influence of voluntary attention in humans, it is probably an evolutionarily ancient, involuntary phenomenon involving alternate selection/suppression of conflicting stimuli, irrespective of the desires of the organism. This raises the question of why an animal might need the capacity to alternate between multiple stimuli, or even to periodically switch among different motor programs [see (Maye et al., 2007) and (Reynolds and Frye, 2007) for *Drosophila* experiments investigating this question]. Viewed from an evolutionary perspective, it is not difficult to imagine that searching for food, mates, or predators could benefit from rapid and flexible disambiguation of conflicting visual, auditory, and olfactory stimuli (or from discriminating figure and ground, in the case of vision). However, switching too fast or too slowly could be disadvantageous in both food/prey and predator scenarios. Similarly, being unable to engage or disengage each alternative with appropriate flexibility could also be disadvantageous, suggesting an interaction between memory mechanisms and rivalry rates.

Selective attention is likely tuned to the rate of change in the environment, and this appears to be the case for miniature brains as well as human brains. Maladaptive behavior apparent in a variety of human cognitive disorders, such as schizophrenia, or in key *Drosophila* mutants proposed as models for these disorders (van Alphen and van Swinderen, 2011), may result in part from a failure to match endogenous attention processes to the pace of a continuously changing and moving environment. Access to ongoing attention-like dynamics in the malleable *Drosophila* brain, by using competing frequency tags, should reveal how brains select and suppress stimuli to maintain appropriate responsiveness levels and behavior in a visually complex environment.

### Conflict of interest statement

The author declares that the research was conducted in the absence of any commercial or financial relationships that could be construed as a potential conflict of interest.
